# Cardiac Involvement of Metastatic Clear Cell Sarcoma: A Multimodality Imaging Report

**DOI:** 10.1161/CIRCIMAGING.121.013902

**Published:** 2022-04-28

**Authors:** Tommaso D’Angelo, Christian Booz, Giuseppe M. Bucolo, Antonino Micari, Ludovica R.M. Lanzafame, Vitali Koch, Alfredo Blandino, Silvio Mazziotti

**Affiliations:** 1 Department of Biomedical Sciences and Morphological and Functional Imaging, University Hospital Messina, Italy (T.D., G.M.B., A.M., L.R.M.L., A.B., S.M.).; 2 Department of Radiology and Nuclear Medicine, Erasmus MC, Rotterdam, Netherlands (T.D.).; 3 Division of Experimental Imaging, Department of Diagnostic and Interventional Radiology, University Hospital Frankfurt, Frankfurt am Main, Germany (C.B., V.K.).

**Keywords:** clear cell sarcoma, heart neoplasms, magnetic resonance imaging, multidetector computed tomography, neoplasm metastasis

A 55-year-old man with history of a metastatic clear cell sarcoma (CCS) came to our observation.

The diagnosis of CCS was originally made in 2005, when the patient developed a palpable mass of the knee, which was surgically removed. After 12 years without any recurrence, a solitary pulmonary nodule was found. The patient underwent lung biopsy with histological confirmation of CCS metastasis. Right pulmonary lobectomy was performed, and the patient underwent chemotherapy with doxorubicin and olaratumab. In 2021, a follow-up total body computed tomography scan showed multiple nodular lesions, mainly in muscles, consistent with a relapse of the disease, and the patient started therapy with pazopanib.

The follow-up computed tomography scan also showed multiple hypodense lesions mainly distributed within the myocardium of the left ventricle (Figure 1A and 1B), which were suspected to be metastatic lesions.

**Figure 1.**
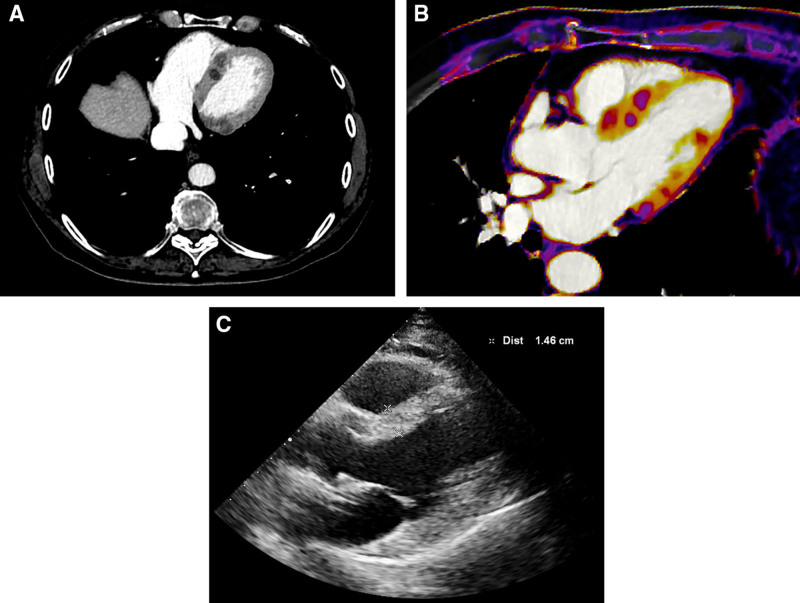
**Computed tomography and transthoracic echocardiography findings. (A**) Axial contrast-enhanced total-body computed tomography image performed during portal venous phase; (**B**) multiplanar image reconstruction along the 3-chamber long-axis view with iodine map overlay. Both images show multiple hypodense lesions within the myocardium, with extensive involvement of inferior and septal segments; (**C**) transthoracic echocardiography image along parasternal long-axis view reveals only septal hypertrophy.

The cardiovascular evaluation was unremarkable. Laboratory tests revealed normal N-terminal pro-brain natriuretic peptide (NT-proBNP) and high-sensitive cardiac troponin (hs-cTn). A 12-lead ECG showed only a sinus bradycardia and a left anterior fascicular block. Transthoracic echocardiography revealed preserved ejection fraction (55%), with interventricular septum hypertrophy and mild reduction of longitudinal function (Figure 1C).

However, because of clinical history and regardless of nonspecific findings, the execution of a cardiac magnetic resonance was suggested.

Cardiac magnetic resonance showed multiple and well-defined nodular lesions with a centimeter maximum size, either intramural and partially exophytic. All lesions were mildly hypertense on T1-weighted images and cine-imaging (Videos S1 and S2), while heterogeneously hyperintense in T2-weighted STIR sequence.

At first-pass imaging, all lesions presented reduced perfusion (Video S3) and heterogeneous late gadolinium enhancement imaging (Figure 2).

**Figure 2.**
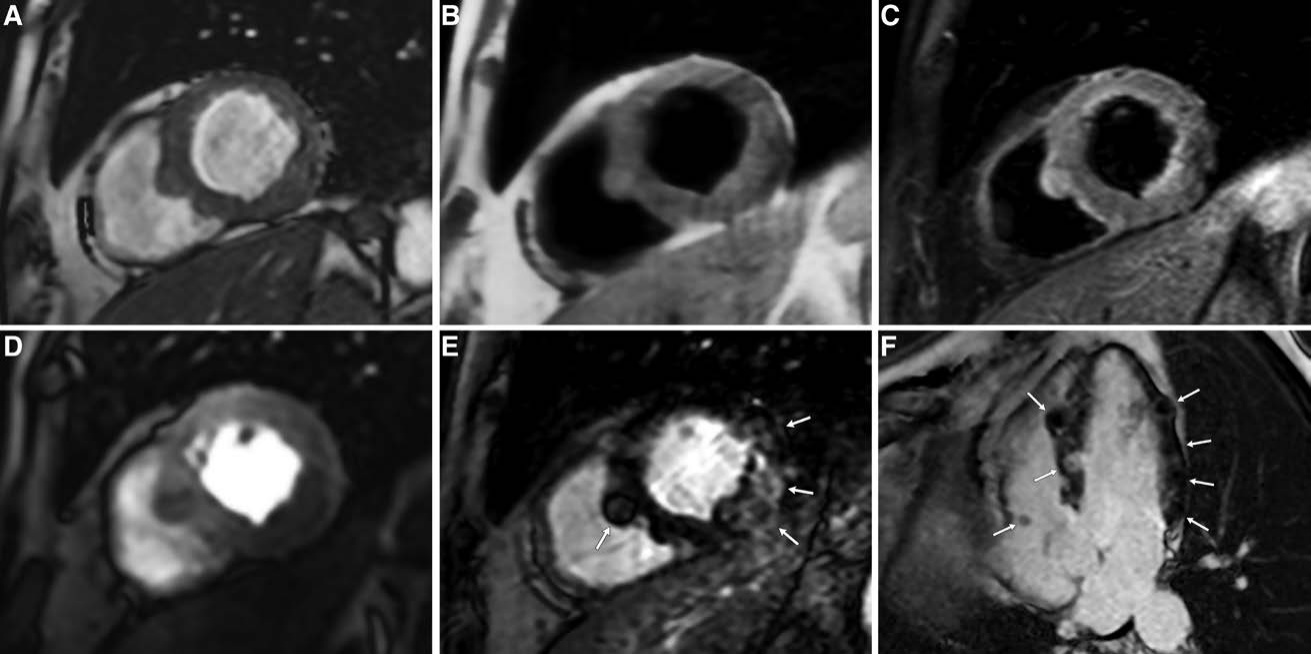
**Cardiac magnetic resonance findings.** Images were performed along the basal short axis (**A–E**) and horizontal long axis (**F**) views. Multiple nodular lesions are visible within the myocardial septal and inferior segments, mildly hypertense on cine-balanced steady state free precession (**A**), and T1-weighted (**B**) sequences and heterogeneously hyperintense to normal myocardium on T2-weighted short-tau inversion recovery sequence (**C**). First-pass perfusion imaging (**D**) shows reduced gadolinium enhancement compared with normal myocardium, mainly involving the inferior wall. Late gadolinium enhancement images (**E** and **F**) show heterogeneous enhancement of the nodular lesions (arrows), along the inferior wall of basal and mid segments, with rosary-beads-like appearance of the moderator band. A small nodular lesion is also visible in the inferior wall of the right atrium.

The majority of lesions involved the inferior and septal segments of the left ventricle, predominantly at the basal and mid slices. Lesions were also found along the moderator band, where they assumed a rosary-beads-like configuration (Figure 2F), and at the inferior wall of the right atrium. Papillary muscles were not involved. A 12-lead ECG performed a few weeks after cardiac magnetic resonance showed the appearance of right bundle branch block (Figure S1).

The imaging characteristics of the nodular lesions were consistent with data reported in literature for CCS. As a result of the variable content of glycogen and melanin, CCS lesions typically seem hyperintense on T1-weighted images and may have heterogeneous signal on T2-weighted and late gadolinium enhancement images.^1^

In fact, despite melanin shorten either T1 and T2, other factors such as cystic degeneration, loose connective tissue between the cellular nests, or cellular morphology may vary signal intensity.^1^

To best of our knowledge, this is the first report of cardiac involvement by metastatic CCS diagnosed antemortem by multimodality imaging.

CCS is a malignant and very rare neoplasm of soft tissues. Its incidence is estimated to account for about 1% of the soft tissue sarcomas.^2^

Although cardiac metastases from CCS are uncommon at the time of the diagnosis, their incidence reaches 25% in autopsy series, with only a small fraction of them diagnosed antemortem.^2,3^

When cardiac involvement has been reported, it mainly manifests with chronic heart failure symptoms. However, this clinical presentation might be mystified by cardiotoxicity induced by doxorubicin, a chemotherapy agent used in the majority of soft tissue sarcomas, including CCS.^3^

A prompt identification of cardiac involvement is crucial in patients suffering from CCS, since the treatment choice may affect the patient’s prognosis. In fact, radiotherapy has shown to increase the survival in case of metastasized soft tissue sarcoma to heart.^4^

Currently, transthoracic echocardiography is considered the modality of choice for first-line imaging assessment of cardiac involvement in patients with suspected metastatic disease.^3,4^ However, ECG and transthoracic echocardiography findings were not specific in our case. Conversely, cardiac magnetic resonance has shown a great potential either to reveal and noninvasively characterize the myocardial lesions, thus allowing for the diagnosis of cardiac metastases from CCS.

## Article Information

### Acknowledgment

We acknowledge Dr C. Buceti for her assistance in providing detailed clinical patient’s data.

### Sources of Funding

None.

### Disclosures

None.

### Supplemental Material

Figure S1

Videos S1–S3

## Supplementary Material
