# Supplementary material for: Cardiac Involvement of Metastatic Clear Cell Sarcoma: A Multimodality Imaging Report
Source: Circ Cardiovasc Imaging. 2022 Apr 28;15(5):e013902. doi: 10.1161/CIRCIMAGING.121.013902 (PMC9112960; doi:10.1161/CIRCIMAGING.121.013902)
Supplement: Supplementary file 1 [file hci-15-e013902-s001.pdf]

SUPPLEMENTAL MATERIAL.

Supplemental Figure.

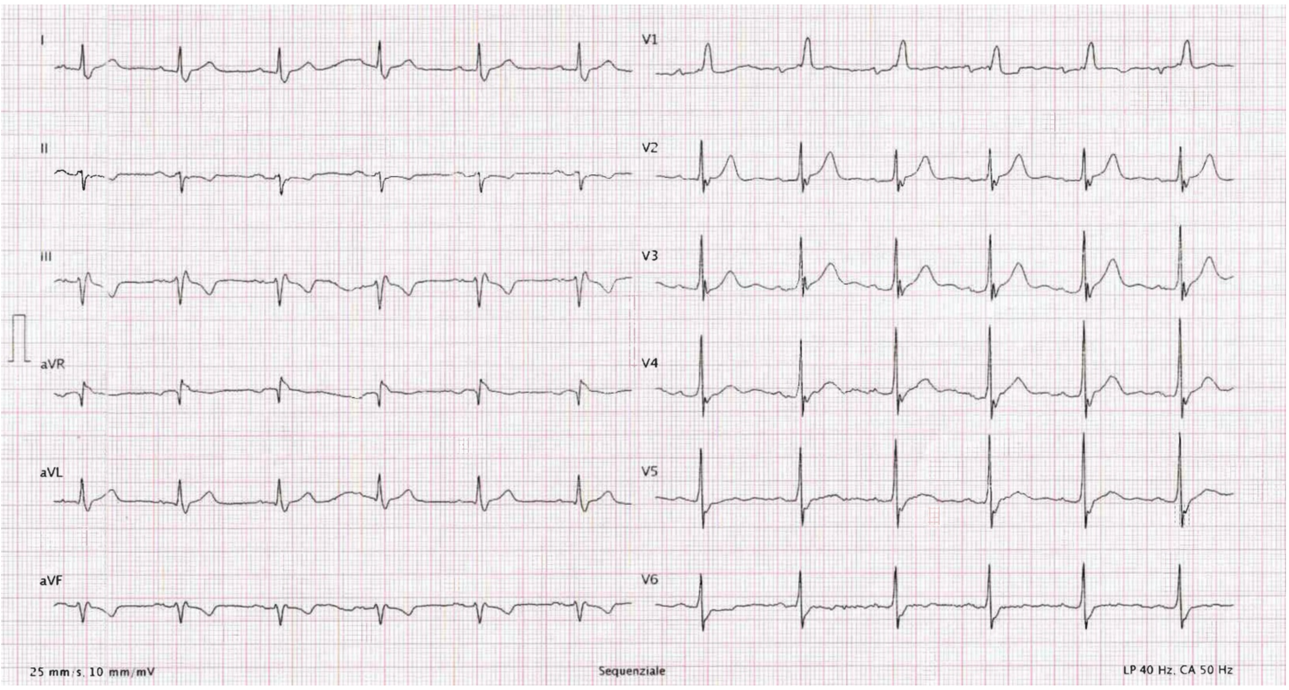

Figure S1

12-lead electrocardiogram (ECG), performed after CMR, showing complete right bundle branch block, and left anterior fascicular block.

Supplemental Video Legends.

Video S1

Cardiac MRI cine-imaging stack performed along the short axis view, showing thickening of septal and inferior myocardial walls, with presence of multiple mildly hyperintense nodular lesions.

Video S2

Cardiac MRI cine-imaging performed along three-chamber view, showing reduced longitudinal function with preserved ejection fraction. Mild aortic regurgitation is also present.

Video S3

First-pass perfusion imaging showing multiple nodular lesions either on basal, mid and apical segments, with reduced gadolinium enhancement.
